# Structure-guided approach to site-specific fluorophore labeling of the *lac* repressor LacI

**DOI:** 10.1371/journal.pone.0198416

**Published:** 2018-06-01

**Authors:** Kalle Kipper, Nadja Eremina, Emil Marklund, Sumera Tubasum, Guanzhong Mao, Laura Christina Lehmann, Johan Elf, Sebastian Deindl

**Affiliations:** Department of Cell and Molecular Biology, Science for Life Laboratory, Uppsala University, Uppsala, Sweden; Weizmann Institute of Science, ISRAEL

## Abstract

The lactose operon repressor protein LacI has long served as a paradigm of the bacterial transcription factors. However, the mechanisms whereby LacI rapidly locates its cognate binding site on the bacterial chromosome are still elusive. Single-molecule fluorescence imaging approaches are well suited for the study of these mechanisms but rely on a functionally compatible fluorescence labeling of LacI. Particularly attractive for protein fluorescence labeling are synthetic fluorophores due to their small size and favorable photophysical characteristics. Synthetic fluorophores are often conjugated to natively occurring cysteine residues using maleimide chemistry. For a site-specific and functionally compatible labeling with maleimide fluorophores, the target protein often needs to be redesigned to remove unwanted native cysteines and to introduce cysteines at locations better suited for fluorophore attachment. Biochemical screens can then be employed to probe for the functional activity of the redesigned protein both before and after dye labeling. Here, we report a mutagenesis-based redesign of LacI to enable a functionally compatible labeling with maleimide fluorophores. To provide an easily accessible labeling site in LacI, we introduced a single cysteine residue at position 28 in the DNA-binding headpiece of LacI and replaced two native cysteines with alanines where derivatization with bulky substituents is known to compromise the protein’s activity. We find that the redesigned LacI retains a robust activity *in vitro* and *in vivo*, provided that the third native cysteine at position 281 is retained in LacI. In a total internal reflection microscopy assay, we observed individual Cy3-labeled LacI molecules bound to immobilized DNA harboring the cognate O_1_ operator sequence, indicating that the dye-labeled LacI is functionally active. We have thus been able to generate a functional fluorescently labeled LacI that can be used to unravel mechanistic details of LacI target search at the single molecule level.

## Introduction

Transcription factors bind to specific DNA sequences at control elements and regulate gene expression by recruitment and regulation of the transcription apparatus. The lactose operon repressor protein LacI from *Escherichia coli* represents one of the structurally [1–5] and biochemically [6–12] best characterized prokaryotic transcription factors and has served as a paradigm for prokaryotic transcription control. Models of facilitated diffusion have been proposed to explain the rapid localization of LacI to its cognate binding sites in the presence of an excess of nonspecific binding sites on the genome [8, 9, 13–15]. Recently, some of these models have been experimentally tested using single-molecule fluorescence microscopy in live cells [16–20]. However, the precise molecular mechanisms by which LacI interacts with DNA while sliding in search for its specific binding sites remain elusive.

*In vitro* single-molecule fluorescence measurements are ideally suited to study such inherently dynamic processes at high spatio-temporal resolution [21, 22]. An often challenging prerequisite for these fluorescence-based imaging approaches is the ability to fluorescently label the protein of interest without compromising its functionality. One of the most widespread methods of protein fluorescence labeling relies on genetically fusing the protein of interest with an autofluorescent protein. For *in vitro* studies, however, synthetic fluorophores often offer a more attractive alternative means of protein labeling. Due to their considerably smaller size compared to the autofluorescent proteins, synthetic fluorophores are less likely to interfere with the folding and function of the protein of interest and offer greater freedom in their placement within the protein structure. Additionally, organic fluorophores are superior to autofluorescent proteins in terms of brightness and photostability, thus allowing to localize the positions of the fluorescently labeled proteins with higher precision and to record longer trajectories in single particle tracking experiments [23, 24].

To enable site-specific labeling with synthetic fluorophores, the protein must contain functional groups at which the fluorophores can be attached. Such functional groups must be accessible to the fluorophore in the context of the native protein structure and must be located in regions where fluorophore attachment does not impair the functionality of the protein. Recent advances in noncanonical amino acid incorporation have made it possible to site-specifically incorporate functional groups for chemoselective protein fluorescence labeling [25–28], representing a promising avenue for single molecule studies. However, the application of this approach is currently hindered by the relatively high cost of the synthetic amino acids, effectively limiting the amount of protein available for fluorescence labeling. As an alternative to the noncanonical amino acid based fluorescence labeling, proteins can be conjugated with synthetic fluorophores at natively occurring cysteine or lysine residues using maleimide or succinimide chemistries, respectively [29]. The latter strategy circumvents the use of expensive synthetic amino acids, relies entirely on the endogenous cellular translation machinery and uses well-established techniques for protein production. Since proteins frequently contain multiple cysteine residues in addition to the one(s) selected for labeling, these additional residues often need to be removed (typically by replacement with alanine) if there is a risk that the fluorophore at those positions would disrupt protein folding or activity. Furthermore, since a native cysteine residue may not always be present at the position most suitable for fluorophore attachment, a cysteine residue must then be introduced at the desired position by site-directed mutagenesis. Since the removal of unwanted native cysteine residues or the introduction of an additional cysteine residue at the labeling position carries the risk of compromising protein function, a careful screening of the replacement variants for their activity is required before a particular variant is chosen for fluorescence labeling. Additionally, the activity of the protein must be analyzed after labeling to ensure that the presence of the fluorophore does not interfere with the functionality of the protein.

The *E*. *coli* lactose operon repressor protein LacI exists *in vivo* as a tetramer of four identical 360 amino acid monomers [6]. The monomer of LacI features an N-terminal DNA-binding domain (residues 1–45) [2, 30–35], followed by a flexible “hinge” region (residues 52–60) [36] that connects the DNA-binding domain to the “core” of LacI (residues 62–332) [1, 2]. The “core” is responsible for the inducer (e.g. lactose, IPTG) binding and dimerization activities of LacI [1–3, 31]. Five principal clusters of amino acids from the “core”, including a region spanning residues 275–290, contribute to the extensive 2200 Å^2^ dimerization interface between LacI monomers [2, 37]. The C-terminal 21 amino acids in LacI (residues 339–360) fold into an amphipatic α-helix that mediates the association of two LacI dimers into a V-shaped tetramer [1, 38] by forming a four-helix bundle involving the C-terminal α-helices from the other three LacI molecules [1, 2, 39]. Tetramerization enables LacI to simultaneously bind two separate operator sites on DNA [7, 40–44], leading to the formation of a looped DNA-LacI complex [12, 45–48]. DNA looping by LacI is required for a full repression of the lac operator *in vivo* [12, 49] and is suggested to facilitate rapid binding site location by LacI in the presence of excess nonspecific DNA [14, 15]. Disruption of the C-terminal coiled-coil interactions by point mutations at specific residues or by deletion of the entire C-terminus abrogates LacI tetramerization, resulting in a dimeric LacI [50–53]. Though separate regions in LacI are responsible for its dimerization and tetramerization, respectively [1, 2], a number of mutations in the monomer-monomer interface more strongly disrupt LacI dimerization in the absence of the tetramerization helix [54, 55], pointing to an interplay between LacI dimerization and tetramerization. The affinity of the dimeric LacI for a single operator site does not markedly differ from the tetramer [48, 50, 52], however, the repressor activity of the dimer *in vivo* is significantly reduced due to its inability bind at two different sites by looping the intervening DNA [48, 56]. Mutations in the dimer-dimer interface result in a monomeric LacI with significantly reduced affinity for DNA [57, 58].

LacI represents a convenient system for a rational redesign *via* site-directed mutagenesis since the substitution tolerance of LacI has been extensively investigated. Using amino acid substitution by nonsense suppression and subsequent *in vivo* screening of functionality, the effects of over 4000 amino acid substitutions at nearly all the positions of LacI have been characterized previously [10, 11, 59, 60]. According to these studies, the “core” of LacI is remarkably substitution-tolerant [10, 11, 59, 60] and even the substitution-sensitive “headpiece” harbors positions where amino acid substitutions minimally affect the functionality of LacI [10, 11, 59, 60]. Moreover, the results of Matthews and coworkers characterizing the reactivity of the cysteine residues in LacI towards sulfhydryl-modifying reagents [61, 62] are of particular relevance for designing LacI variants for labeling with maleimide fluorophores. Coupled with the structural information on LacI in the apo form and in complex with ligands [1–4, 37, 38], this genetic and biochemical information is instrumental in the design of LacI variants for small molecule fluorescence labeling.

In this study, we present a structure-guided cysteine mutagenesis to generate dimeric LacI variants for maleimide-based fluorophore labeling. A functionally active, dimeric, and organic dye-labeled LacI would represent an experimentally tractable model system to study a number of important questions related to the molecular mechanisms of transcription factor target search at the single-molecule level. The LacI variants were screened for functionality *in vitro* using size exclusion chromatography, dynamic light scattering, and gel mobility shift analyses. Moreover, we employed a β-galactosidase assay to assess the *in vivo* activities of the variants. Based on this strategy, we selected three single-cysteine mutants of LacI that retained near-wild type activity. Single-molecule fluorescence imaging of a representative LacI mutant reveals binding to the cognate target DNA sequence upon fluorescence labeling with Cy3.

## Materials and methods

### Plasmids

A C-terminally 6xHis-tagged variant of LacI in the arabinose-inducible plasmid pBAD24 was used as a template for the introduction of amino acid substitutions into LacI. In this construct, the C-terminal tetramerization helix of LacI has been replaced with a 6xHis-purification tag while the rest of the coding sequence is that of WT LacI. Single amino acid substitutions were introduced into LacI using the Quick Change mutagenesis kit. The identities of the resulting LacI variants were verified by sequencing using the sequencing primers pBAD_fwd_primer (ATGCCATAGCATTTTTATCC) and pBAD_rev_primer (CCTGATACAGATTAAATC).

### LacI expression and purification

The LacI variants were expressed in *E*.*coli* TOP10. Briefly, overnight cultures of *E*. *coli* TOP10 cells transformed with one of the pBAD24-based constructs were diluted into 800 mL LB medium containing 100 μg/mL ampicillin and grown at 30°C under constant agitation. The expression of LacI was induced at OD_600_ = 0.45 by the addition of L-arabinose to a final concentration of 1.5 mg/mL and the incubation was continued at 30°C for 4 h. The cells were subsequently harvested by centrifugation at 6,000 x g/room temperature for 20 min. After removing the supernatant the cells were resuspended in 25 mL of buffer A (20 mM Tris-HCl, pH 8, 500 mM NaCl, 20 mM imidazole, 5 mM β-mercaptoethanol, and 10% glycerol) supplemented with 4 EDTA-free protease inhibitor pills (Roche) and lyzed on ice (One Shot Cell Disrupter, Constant Systems Ltd). The resulting lysate was first clarified by centrifugation at 16,000 x g/4°C for 1 h, followed by filtering the supernatant through a a 0.2 μm cellulose acetate membrane filter.

All subsequent purification steps were carried out at 4°C on an ÄKTA FPLC system (GE Healthcare). The clarified lysate was applied (at a flow rate of 0.5 mL/min) to a 5 mL HisTrap HP affinity purification column (GE Healthcare) equilibrated with buffer A and the column was washed with 10 column volumes of the same buffer at a flow rate of 1 mL/min until reaching a stable baseline in A_280_ absorbance. Bound protein was eluted from the column in buffer B (20 mM Tris-HCl, pH 8, 500 mM NaCl, 500 mM imidazole, 5 mM β-mercaptoethanol and 10% glycerol) at a flow rate of 1 mL/min. Eluted fractions were analyzed on a 4–20% Mini-PROTEAN TGX SDS-PAGE (Biorad) gel, and LacI containing fractions were pooled and concentrated to a volume of 1 mL using a 10 kDa MW cut-off Sartorius Vivaspin Turbo concentrator.

The affinity-purified LacI preparation was subsequently applied (at a flow rate of 1 mL/min) to a HiLoad 16/600 Superdex 200 size exclusion column equilibrated with buffer C (200 mM Tris-HCl, pH 7.5, 200 mM KCl, 1 mM EDTA and 0.5 mM TCEP) and eluted with 1.5 column volumes of the same buffer at a flow rate of 1 mL/min. Only one protein peak was observed during the elution. The peak fractions were analyzed on a 4–20% Mini-PROTEAN TGX SDS-PAGE (Biorad) gel, pooled and concentrated to a volume of 1 mL using a 10 kDa MW cut-off Sartorius Vivaspin Turbo concentrator. The amount of protein recovered at this stage varied between 2 mg and 45 mg depending on the LacI variant. The concentrated LacI protein was divided into 20 μl aliquots, flash-frozen in liquid nitrogen and stored at -80°C.

### Preparation of dsDNA substrates

Double-stranded DNA oligonucleotides were generated by PCR-amplification using a double-stranded gBlock DNA (IDT DNA) harboring the O_1_ operator sequence (AATTGTGAGCGGATAACAATT) as the template. A 5’-Cy5 or 5’-biotin moiety was introduced using primers covalently labeled (IDT DNA) with Cy5 (for the EMSA experiment) or 5’-biotin (for the TIRF experiment) *via* a flexible linker.

### Estimating the affinity of the LacI variants for the O_1_ operator by an electrophoretic mobility shift assay (EMSA)

LacI-O_1_ operator complexes were formed by incubating 1 nM 5’-Cy5-labeled dsDNA oligonucleotide harboring the O_1_ operator sequence with 0.5–100 nM of one of the unlabeled LacI variants in binding buffer (10 mM Tris pH 7.5, 50 mM NaCl, 3 mM MgCl_2_, 1 mM TCEP, 0.1 mg/mL BSA, 5% glycerol) at room temperature for 1 h. LacI-operator complexes were resolved by native PAGE on an 8% polyacrylamide gel (BioRad) and visualized on a ChemiDoc Imaging System (BioRad) using a 650 nm excitation light for Cy5. The intensities of the bands corresponding to the LacI-operator complex were quantified in ImageJ image processing software and further analyzed as described below. We used the Cy5-labeled O_1_ operator DNA at 1 nM concentration, within the range of the K_d_ values of 0.74 and 1.60 nM estimated for the operator binding of dimeric LacIs missing the 18 or 32 C-terminal amino acids, respectively [51]. Our LacI variants lack 22 native amino acids from the C-terminus and their operator affinities are thus likely to be similar to the ones reported in [51]. As the operator concentration in our assay is close to the K_d_ of the operator-LacI dimer complex, the value of K_d_ must be estimated by fitting the binding data to the following equation

$$[RL]=\frac{([R_{T}]+[L_{T}]+K_{d})-\sqrt{{\left[[R_{T}]+[L_{T}]+K_{d}\right]}^{2}-4[R_{T}][L_{T}]}}{2},$$

where [L_T_] is the total concentration of LacI, [R_T_] is the total concentration of the Cy5-labeled dsDNA oligonucleotide and K_d_ is the dissociation constant. This equation is a full quadratic expansion of the binding polynomial obtained when solving for the concentration of the complex RL in a reversible binding reaction of the type R + L ↔ RL under conditions where the concentration of one of the binding partners is near the K_d_ of the complex [63–65]. We note that above equation does not take into account the cooperativity observed for dimeric LacI binding to DNA that arises from the LacI monomer-dimer equilibrium [52].

### Analytical size exclusion chromatography

For analytical size exclusion chromatography, samples were injected onto a HiLoad 16/600 Superdex 200 column. Each run was performed at a flow rate of 0.2 mL/min with buffer C. The calibration of the column was carried out with a Gel Filtration Markers Kit (Sigma-Aldrich) according to manufacturer’s instructions.

### Dynamic light scattering (DLS)

5 μl samples were measured in a BladeCell cuvette (Avid Nano) using a W130i DLS system (Avid Nano) at 4°C and protein concentrations of 12.1 mg/mL, 12.8 mg/mL, and 12.5 mg/mL for LacI^ΔCterm^, LacI^Cys-Lite^, and LacI^Dimer^, respectively.

### Labeling LacI with Cy3-maleimide

Labeling of LacI with Cy3-maleimide was performed in a buffer containing 100 mM potassium phosphate pH 7.0, 150 mM NaCl, and 5 mM EDTA. The labeling buffer was degassed by sonication for 15 min (VWR ultrasonic cleaner) and kept on ice for 5 min. To 300 μL of the labeling buffer TCEP and LacI were added to final concentrations of 100 μM and 10 μM, respectively, followed by a 30 min incubation of the mixture at room temperature in the dark. The 300 μL LacI sample was subsequently mixed with 30 μL of Cy3-maleimide dissolved in DMF (final concentration of Cy3-maleimide in the resulting 330 μL: 640μM) and incubated at room temperature for 2 h, followed by an overnight incubation at 4^o^ C. The labeling mixture was kept in the dark throughout the dye treatment. The labeling reaction was quenched by the addition of β-mercaptoethanol to a final concentration of 4.5 mM and excess unreacted dye was removed by applying the labeling mixture (in 150 μL aliquots) through a spin column containing 300 μL Pierce Dye Removal Resin in two repeated purification steps following the manufacturer’s instructions. Cy3-labeled LacI was dialyzed into buffer C containing 50% glycerol overnight at 4^o^ C in a Pierce 20 kDa Mw cut-off Slide-a-lyzer dialysis unit, aliquoted and stored at– 20^o^ C. The labeling efficiency ([Cy3]/[LacI dimer] = 57%), was calculated by separately determining the LacI and Cy3 fluorophore molar concentrations based on absorbance measurements at 280 nm and 550 nm, respectively.

### Beta-galactosidase assay

The LacI deletion strain ΔLacI devoid of the chromosomal LacI locus was constructed by λ Red recombination of a PCR fragment harbouring a chloramphenicol (cam) resistance cassette into the strain BW25993 [MG1655 *Δ(araD-araB) 567 rph-1 Δ(rhaD-rhaB) 568 hsdR514*] containing the pKD46 plasmid [66]. The cam-cassette was subsequently removed by FLP recombinase, leaving FRT scars [66]. The ΔLacI strain was transformed with the pBAD24-based constructs harboring the LacI variants and the cells were grown overnight in LB in the presence of 0.1 mg/mL ampicillin at 37^o^ C. The overnight cultures were diluted to an initial density of ΔOD_600_ = 0.08 in 5 mL LB containing 0.1 mg/mL ampicillin and 0.02% (v/v) L-arabinose to induce the expression of the plasmid-borne LacI variants. The cells were grown with constant shaking at 37^o^ C to a density of ΔOD_600_ = 0.7–0.8. At that point the cultures were diluted to a ΔOD_600_ = 0.4 with fresh LB medium and each culture was then divided into two 2.5 mL aliquots. One of the pair of aliquots was mixed with IPTG to a final concentration of 1 mM while the other aliquot was mixed with an equal volume (2 μL) of water. The cultures were incubated at 37^o^ C for 2.5 h and then diluted with LB to ΔOD_600_ = 0.3. From each diluted culture 0.4 mL aliquots were withdrawn and mixed with an equal volume of a permeabilization solution (0.6 mg/mL chloramphenicol, 0.05% (v/v) sodium dodecyl sulphate, 50 mM β-mercaptoethanol, 2% (v/v) chloroform, 10 mM KCl, and 1 mM MgCl_2_ in 0.1 M NaH_2_PO_4_/Na_2_HPO_4_ buffer, pH 7). The samples were incubated for 5 min at 30^o^ C in a water bath. Next, the samples were mixed with 2-nitrophenyl-β-D-galactopyranoside (ONPG) to a final concentration of 0.7 g/L and incubated at 28°C in a water bath for 1.5 min. The reactions were stopped by the addition of Na_2_CO_3_ to a final concentration of 0.29 M. In the control reactions (“blanks”), Na_2_CO_3_ was added (to a final concentration of 0.29 M) to the permeabilized cells expressing a given LacI variant prior to the ONPG addition. The subsequent processing of the controls was identical to the samples incubated with ONPG for 1.5 min prior to Na_2_CO_3_ addition. The samples were centrifuged at 16 200 x g at room temperature for 3 min and the release of 2-nitrophenol was measured in the supernatant spectrophotometrically at 420 nm. The A_420_ readings from the control samples were subtracted from the A_420_ readings of the samples incubated with ONPG for 1.5 min prior to Na_2_CO_3_ addition. The activity of β-galactosidase was expressed in Miller units according to 1000*ΔA_420_/[ΔOD_600_*t*V] [67], where ΔA_420_ is the sample optical density measured at 420 nm, ΔOD_600_ is the culture density at the beginning of the assay, t is the time (in minutes) of sample incubation with 2-nitrophenyl-β-D-galactopyranoside, and V is the culture volume (0.4 mL).

### Total internal reflection fluorescence (TIRF) microscopy and single-molecule imaging of Cy3-LacI

Biotinylated (but not fluorophore-labeled) double-stranded DNA was surface-anchored on poly[ethylene glycol]-coated quartz microscope slides through biotin-streptavidin linkage. Binding of LacI was observed at room temperature upon infusing the sample chamber with the imaging buffer supplemented with 500 pM Cy3-LacI using a syringe pump (Harvard Apparatus). DNA-bound and therefore immobilized Cy3-LacI was excited with a 532 nm Nd:YAG laser (Cobolt) and fluorescence emission from Cy3 was detected using a prism-type TIRF microscope, filtered with a 585 nm bandpass filter (Chroma Technology), and imaged onto a CCD camera (Andor BRAND iXon^EM^+888 512 x 512). The imaging buffer contained 100 mM phosphate buffer, pH 7.4, 1 mM NaCl, 0.05 mM EDTA, 3 mM MgCl_2_, 0.01% Tween, 1 mM β-mercaptoethanol, and an oxygen scavenging system (10% glucose, 800 μg mL^-1^ glucose oxidase, 40 μg mL^-1^ catalase) to reduce photobleaching, 2 mM Trolox (Sigma) to reduce photoblinking of the dyes [68] and 0.1 mg mL^-1^ BSA (Promega).

## Results and discussion

### Native cysteines in LacI are ill-suited for fluorophore attachment

In this study, various positions in LacI were subjected to mutagenesis to generate functionally active variants for a site-specific fluorophore labeling as described in the following sections. All variants were constructed based on a LacI where the C-terminal tetramerization helix (residues 339–360) is replaced with a 6xHis affinity tag to facilitate the purification of the labeled protein from excess fluorophore. This initial LacI variant harbouring the 6xHis tag but carrying an otherwise wild-type amino acid sequence will be referred to as “LacI^ΔCterm^” (Table 1). Due to the replacement of the tetramerization helix with the 6xHis tag none of the LacI variants–including “LacI^ΔCterm^”–is expected to form tetramers [50–53]. However, this lack of tetramerization in our LacI variants does not preclude the identification of fluorophore attachment sites in LacI that are compatible with its dimerization and DNA binding activities. Once suitable labeling positions have been established in the dimeric LacI, the tetramerization helix could be easily reinstalled.

**Table 1:** A summary of the LacI variants used in this study. In all LacI variants the native 22 C-terminal amino acids have been replaced with a 6xHis purification tag. LacI^ΔCterm^ is wild-type with respect to the protein sequence outside of the 22 C-terminal amino acids and served as the basis for the construction of the remaining LacI variants.

Name of the variant	Mutations outside of tetramerization helix	Presence of tetramerization helix [residues 339–360]
LacI^ΔCterm^	None	Absent
LacI^Cys-Lite^	C107A, C140A, C281A	Absent
LacI^Dimer^	C107A, C140A	Absent
LacI^Dimer^—Y12C	C107A, C140A, Y12C	Absent
LacI^Dimer^—S28C	C107A, C140A, S28C	Absent
LacI^Dimer^—L62C	C107A, C140A, L62C	Absent

**Fig 1:**
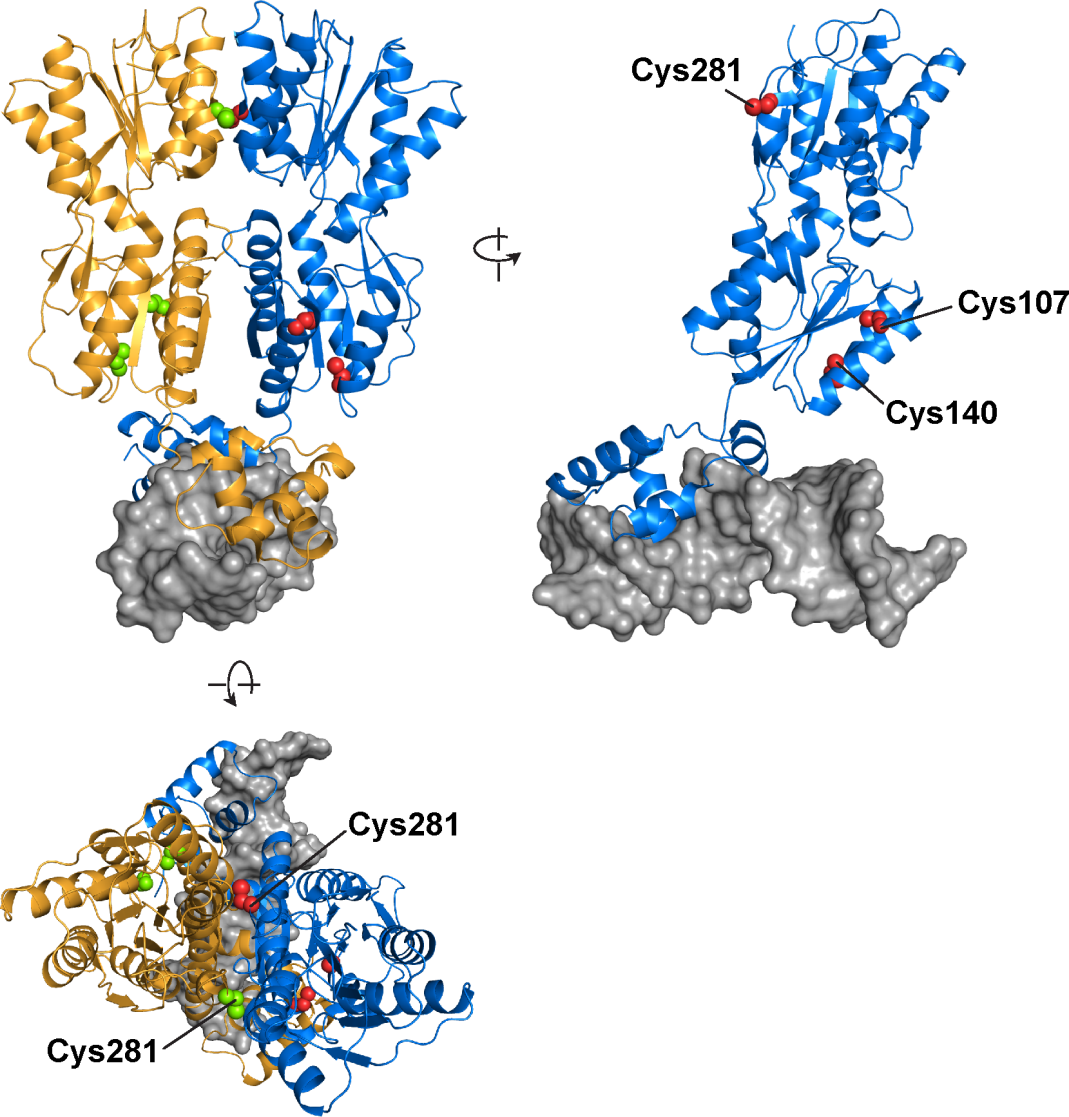
Location of the native cysteine residues of LacI. Cartoon representation of the LacI dimer-operator complex structure (PDB ID: 1EFA) [3]. The two LacI monomers are shown in orange and blue with their native cysteines highlighted as green and red spheres, respectively. The operator DNA is shown as a grey surface. The structure of the LacI-operator complex was rendered in Pymol (PyMOL Molecular Graphics System, Version 2.0 Schrödinger, LLC).

We initiated the design of LacI variants for the labeling with maleimide fluorophores by first analyzing the suitability of the three native cysteines (Cys107, Cys140, Cys281) as sites of fluorophore attachment. Among these, cysteines Cys107 and Cys140 appear buried within the “core” of LacI (Fig 1), suggesting that they may not constitute readily available fluorophore attachment sites. However, despite their internal location, these residues have been shown to be easily derivatized with sulfhydryl-specific modifying agents [61]. Importantly, while the derivatization of cysteines Cys107 and Cys140 with less bulky maleimide reagents only minimally perturbed LacI activity, attachment of a bulkier N-[3-pyrene]-maleimide fluorescent probe (similar in size to commonly used fluorescent dyes) was shown to result in a 50% loss of LacI operator binding activity [61]. We therefore chose to replace cysteines Cys107 and Cys140 with alanines to avoid a potential loss of LacI activity upon labeling with maleimide fluorophores. In replacing Cys107 and Cys140, we were additionally guided by previous substitution tolerance analyses indicating that a Cys-to-Ala substitution at positions 107 and 140 does not markedly impair the activity of LacI *in vivo* [10, 11]. The remaining third native cysteine at position 281 is located in the monomer-monomer interface of the LacI dimer. Although the interfacial location of Cys281 has been shown to render it considerably less reactive to sulfhydryl-modifying reagents when compared to Cys107 and Cys140 [61, 62], we reasoned that even an occasional derivatization of Cys281 with a bulky substituent could compromise the function of LacI as shown previously for a derivatization of LacI [62]. In our initial design we therefore replaced Cys281 with alanine alongside Cys107 and Cys140. In replacing Cys281 with alanine we were encouraged by previous studies showing that a Cys-to-Ala substitution at position 281 of LacI is well-tolerated with respect to both operator and inducer binding [10, 11, 58, 69]. Our initial design thus resulted in a completely cysteine-free variant of LacI that we refer to as “LacI^Cys-Lite^” (Table 1) and that we intended to utilize as a starting point for the introduction of cysteine residues in locations more suitable for fluorophore attachment.

### LacI^Cys-Lite^ loses dimerization and DNA binding abilities

**Fig 2:**
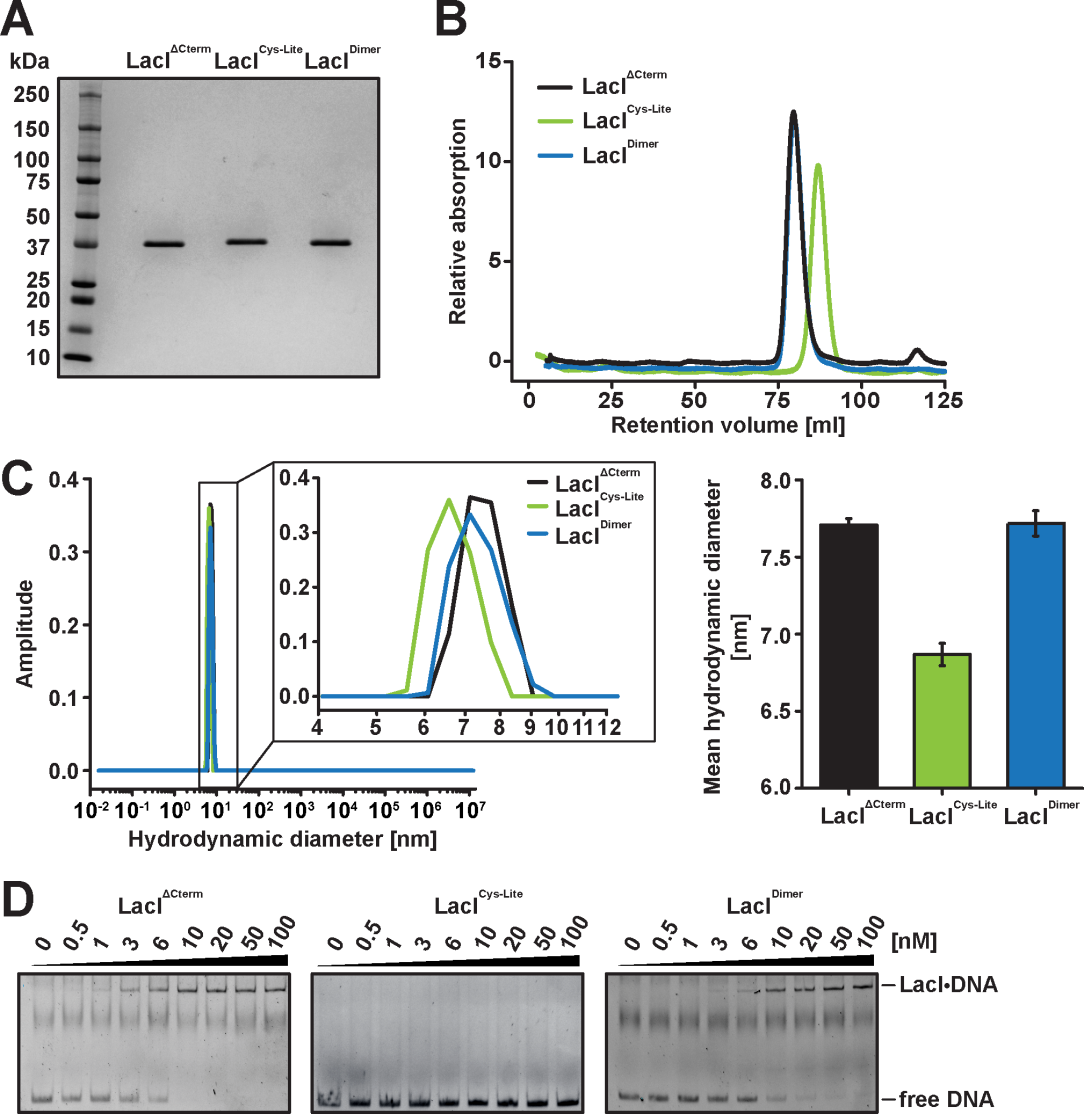
Analysis of the dimerization and DNA binding activities of the LacI^Cys-Lite^ and LacI^Dim^ variants. (A) The C-terminally 6xHis-tagged LacI variants were purified by Ni-agarose affinity chromatography followed by a size exclusion chromatography on Superdex 200. The homogeneity of the purified LacI variants was analyzed on a 4–20% denaturing polyacrylamide gel. The protein was visualized by staining the gel with Coomassie Brilliant Blue. (B) The molecular weights of the LacI variants were estimated by analytical gel filtration on a HiLoad 16/600 Superdex 200 size exclusion column (see S1 Fig for the calibration curve). (C) The mean hydrodynamic diameters of the LacI variants were estimated by dynamic light scattering using a W130i DLS system at 4°C. Error bars represent SEM (*N* = 4 independent experiments with 10 technical replicates each). (D) The operator binding activities of the LacI variants were analyzed in an electrophoretic mobility shift assay. A constant amount of a 5’-Cy5-labeled dsDNA harboring the O_1_ operator sequence was incubated with increasing concentrations of one of each of the LacI variants at room temperature for 1 h. The LacI-operator complexes were resolved on an 8% native polyacrylamide gel and the free and LacI-bound DNA in the gels was visualized by Cy5 fluorescence.

Variants of LacI bearing Cys-to-Ala substitutions at all three native cysteine positions have previously been used in studies of the LacI-induced DNA looping [70] and LacI diffusion on flow-stretched DNA [71], indicating that such native cysteine-free variants are functional. As opposed to the LacI variants in these studies, our “LacI^Cys-Lite^” lacks the C-terminal tetramerization helix. Since the effects of mutations affecting LacI dimerization are more pronounced in the absence of tetramerization [54, 55], we tested the activity of the “LacI^Cys-Lite^” variant prior to the site-specific insertion of non-native cysteine residues (Fig 2) by examining the ability of “LacI^Cys-Lite^” to dimerize and bind to the operator DNA. We subjected LacI^Cys-Lite^ to analytical size exclusion chromatography using a Superdex S200 gel filtration column previously calibrated with proteins of known molecular weight (Fig 2B and S1 Fig). Strikingly, LacI^Cys-Lite^ eluted from the column at a retention volume of 87.3 mL that corresponds to an estimated molecular weight of 36.7 ± 13.3 kDa, consistent with a monomer in solution (Fig 2B). In contrast, the LacI^ΔCterm^ variant that lacks the teramerization helix similarly to “LacI^Cys-Lite^” but retains all native cysteines eluted at 79.5 mL (estimated molecular weight: 69.1 ± 25.0 kDa), consistent with its being a dimer (Fig 2B). Furthermore, dynamic light scattering (DLS) measurements (Fig 2C) yielded hydrodynamic diameters of 7.71 ± 0.04 or 6.87 ± 0.07 nm for LacI^ΔCterm^ or LacI^Cys-Lite^, consistent with a dimer or monomer, respectively. The monomeric state of LacI^Cys-Lite^ indicates that at least one of the three native cysteines in LacI cannot be replaced without abolishing the dimerization ability of the protein. Next, we carried out gel mobility shift assays to examine LacI binding to a double-stranded DNA oligonucleotide carrying the native O_1_ operator sequence. With LacI^ΔCterm^, complex formation was observed at ~3 nM protein concentration (Fig 2D). In contrast, LacI^Cys-Lite^ displayed negligible complex formation even at the highest protein concentration (100 nM) used (Fig 2D). Taken together, our data indicate that at least one of the native cysteines in LacI is essential for the functionality of the protein in the absence of the ability to tetramerize and must therefore be retained in the labeling variants.

### Cysteine residue at position 281 is essential for LacI dimerization and DNA binding

Next, we sought to identify which of the three Cys-to-Ala replacements in LacI^Cys-Lite^ might be responsible for its loss of dimer formation and DNA binding ability. Given its central location in the monomer-monomer interface of the LacI dimer, the cysteine at position 281 represents one plausible candidate (Fig 1) [2, 53, 58]. We therefore reintroduced Cys281 and analyzed the dimerization and DNA binding abilities of the resulting single-cysteine variant of LacI. Importantly, this LacI variant formed a dimer in solution as judged by gel filtration and DLS analyses (Fig 2B and 2C), indicating that the presence of a single cysteine at position 281 is sufficient for LacI dimerization. In the following, we therefore refer to this single-cysteine variant as LacI^Dimer^. Moreover, LacI^Dimer^ was able to bind to the O_1_ operator sequence, in stark contrast to the cysteine-free LacI^Cys-Lite^ variant (Fig 2D). Thus, out of the three native cysteines only the cysteine at position 281 appears essential for the functionality of LacI and must be retained in the labeling variants. While in principle, retaining Cys281 carries the risk of unwanted attachment of a bulky substituent at this position during labeling, previous studies have shown Cys281 to be poorly accessible to cysteine-reactive probes in wild-type LacI [61, 62, 72], indicating that this residue is buried in the monomer-monomer interface [73]. We therefore reasoned that the accessibility of the fluorophore to this residue will be negligible in the dimeric form of LacI prevailing under our labeling conditions where the concentration of LacI (10 μM) is in a 130-fold excess of the monomer-dimer equilibrium constant of 7.7 x 10^−8^ M estimated for a variant of LacI incapable of forming tetramers [52].

### A structure and genetics guided introduction of cysteines into LacI for fluorescence labeling

**Fig 3:**
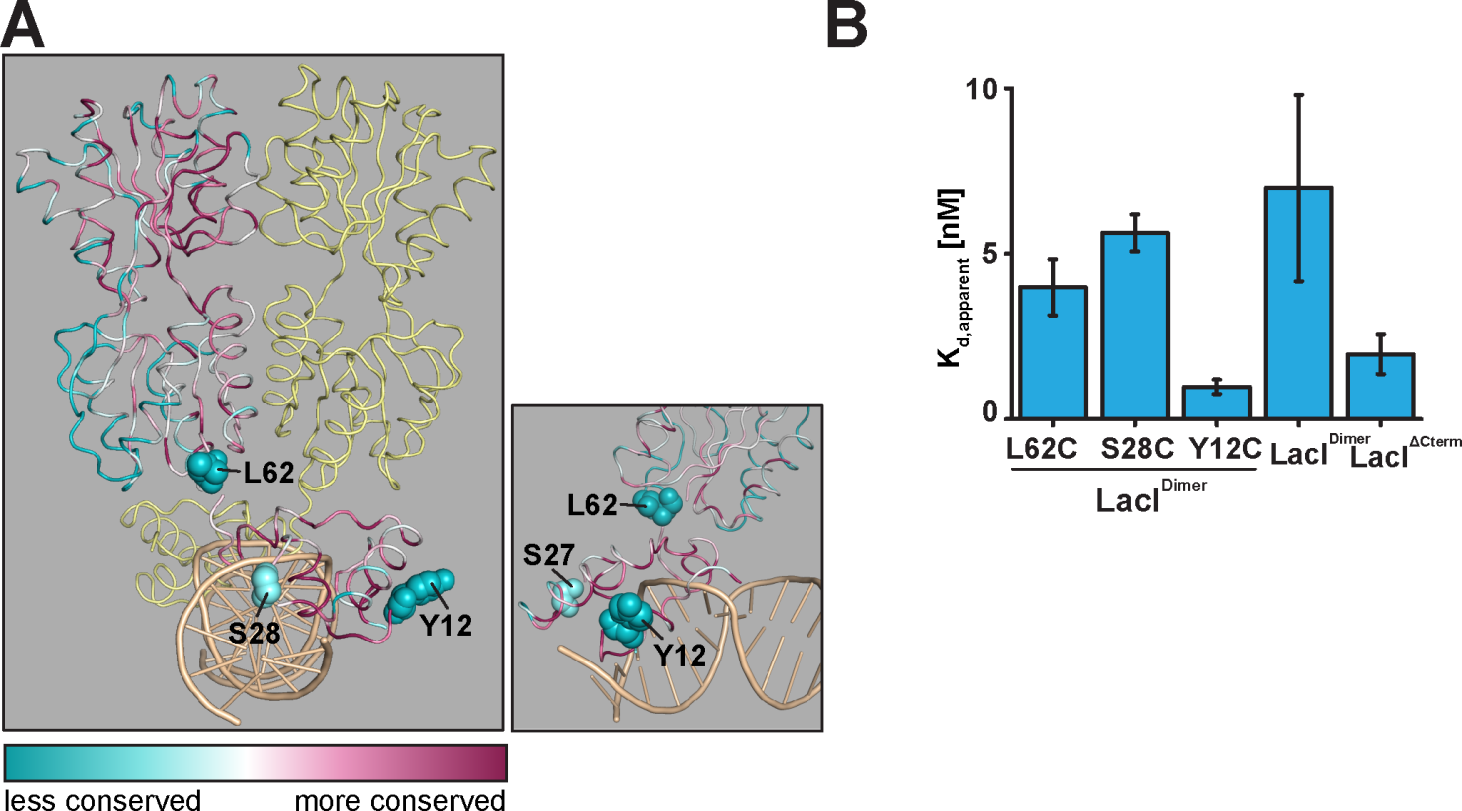
Introduction of additional cysteines into LacI^Dimer^ for fluorescence labeling. (A). The positions where additional cysteines were introduced for labeling are shown as spheres in the structure of the wild-type LacI dimer-operator complex (PDB ID: 1EFA) [3] color-coded for residue conservation. The residue conservation score from a multiple sequence alignment was mapped onto the structure of LacI using the Consurf web server (at http://consurf.tau.ac.il) [74,75]. Insert: a close-up view of the positions for cysteine introduction. (B) Affinities of the LacI variants LacI^Dimer^-Y12C, -S28C, and -L62C for the O_1_ operator sequence were analyzed in an electrophoretic mobility shift assay performed as in Fig 2C. The apparent K_d_ values of the LacI-operator O_1_ complexes were estimated by a nonlinear regression of the LacI-DNA band intensity *versus* LacI concentration curves (S2 Fig) as described in the Methods section. Error bars represent SEM (*N* = 3 independent experiments).

Based on LacI^Dimer^ as a functional chassis, we proceeded to introduce single cysteine residues at positions more suitable for fluorophore attachment than those of the native cysteines. For this, we selected positions 12, 28 and 62 in LacI (Fig 3A) based on their a) solvent exposure as revealed by an inspection of available structural information on LacI, b) lack of involvement in DNA or inducer binding and c) low residue conservation score in a multiple sequence alignment. In selecting these positions for cysteine insertion, we were additionally guided by previous work that suggested tolerance towards amino acid substitutions at these positions [10, 11, 59]. The respective LacI variants will be referred to as LacI^Dimer^ -Y12C, -S28C, and -L62C in the following text. In each case, the position of cysteine insertion is distal to functionally important regions of LacI and exhibits a low residue conservation score. Consistent with this, all three cysteine variants retained robust DNA binding with apparent dissociation constants (K_d_) similar to that of LacI^ΔCterm^ (Fig 3B).

**Fig 4:**
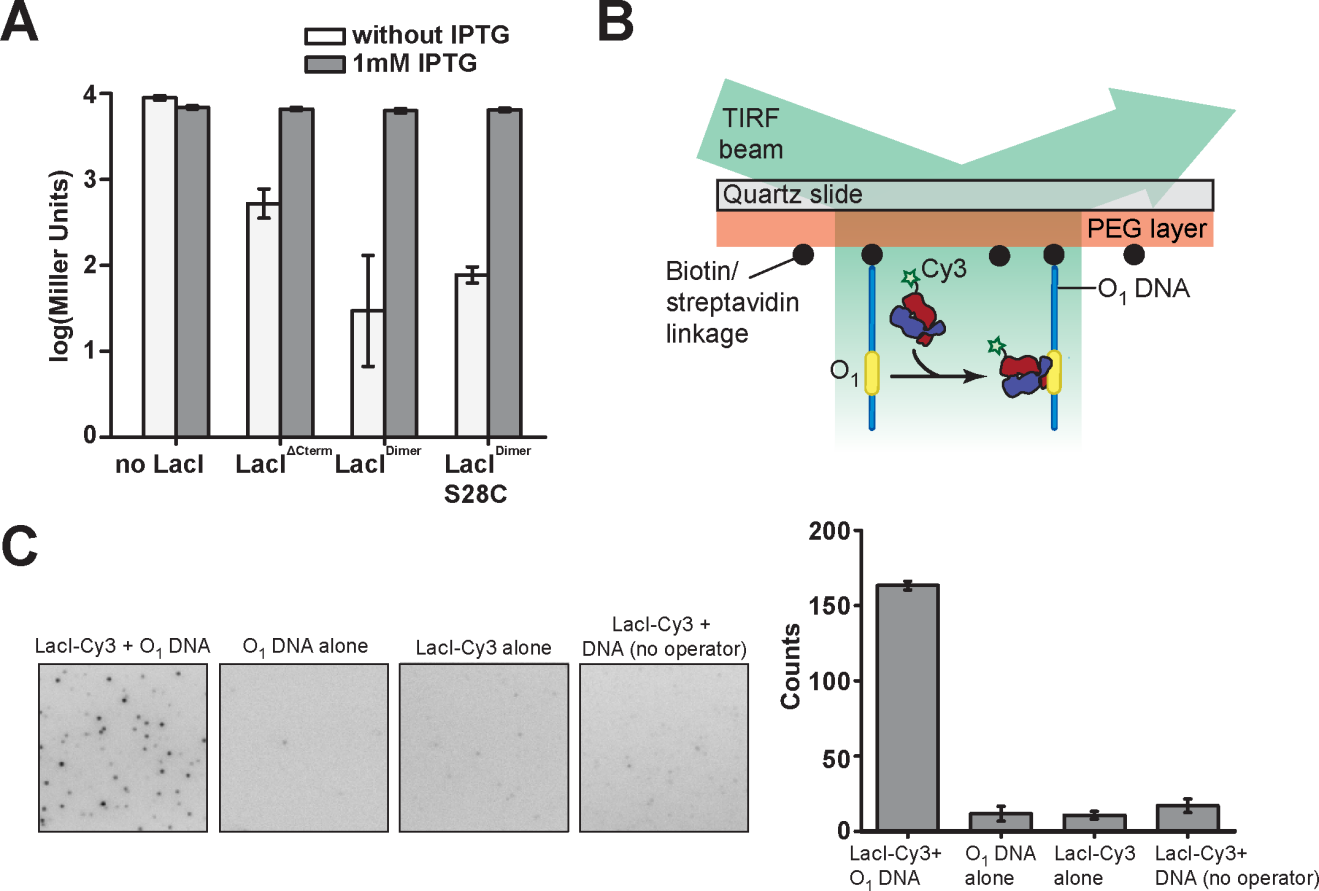
Analysis of the functionality of the LacI variants LacI^Dimer^- Y12C, -S28C, and -L62C. (A) A β-galactosidase assay of the repressor activities of the LacI variants in a derivative of *E*. *coli* strain BW25993 lacking endogenous LacI. The LacI variants were expressed from pBAD24 in the presence of 0.02% L-arabinose at 37° C. β-galactosidase expression was induced with 1 mM IPTG for 2.5 h at 37° C. Y-axis: log_10_ of Miller units. Miller units were calculated as: 1000*ΔA4_20_/[ΔOD_600_*t*V] where ΔA_420_ is the sample optical density measured at 420 nm, ΔOD_600_ is the culture density at the beginning of the assay (ΔOD_600_ = 0.3), t is the time of sample incubation with 2-nitrophenyl-β-D-galactopyranoside (1.5 min) and V is the culture volume (0.4 mL). Each sample was measured in triplicate. (B) Schematic for the single-molecule detection of individual Cy3-labeled LacI^Dimer^-S28C (LacI-Cy3) binding to surface-immobilized (biotin-streptavidin linkage) dsDNA harboring the O_1_ operator (O_1_ DNA) sequence. (C) Left: Distinct fluorescence peaks indicating binding could only be detected (areas of 150 x 150 pixels are shown) in the presence of both LacI-Cy3 as well as a specific operator sequence. Right: Quantification of the number of fluorescent peaks above background recorded in an area of 300 x 300 pixels, determined in triplicates for each condition.

For proof-of-principle, we arbitrarily selected one of these sites, LacI^Dimer^ S28C, for further characterization, fluorophore labeling, and fluorescence microscopy. In order to examine the *in vivo* activity of LacI^Dimer^ S28C, we carried out ß-galactosidase assays (Fig 4A). LacI^Dimer^ exhibited a wild type-like response to the inducer IPTG, indicating that both the Cys-to-Ala substitutions at positions 107 and 140 in LacI are compatible with its repressor activity *in vivo*. Moreover, consistent with the ability of LacI^Dimer^ S28C to bind to the operator site in *vitro* (Fig 3B), the S28C mutation did not affect repressor activity of LacI in *in vivo* (Fig 4A). Next, we site-specifically labeled LacI^Dimer^ S28C at position 28 with maleimide-Cy3 to obtain Cy3-labeled LacI, referred to as LacI-Cy3. We imaged surface-immobilized double-stranded DNA containing an O_1_ operator (O_1_ DNA) site using total internal reflection fluorescence (TIRF) microscopy (Fig 4B). Notably, in the presence of both LacI-Cy3 and O_1_ DNA but not with O_1_ DNA or LacI-Cy3 by itself, we observed discrete fluorescence peaks indicating immobilized, DNA-bound LacI-Cy3 (Fig 4C). We note that we cannot formally rule out the possibility that fluorophore labeling reduced the binding affinity of LacI-Cy3 in comparison to the unlabeled LacI^Dimer^ S28C. However, even at concentrations as low as 500 pM LacI-Cy3, we observed a large number of binding events (Fig 4C, right). The K_d_ value for the fluorophore-labeled LacI species is therefore most likely not substantially larger than the K_d_ value of 5.63 ± 0.56 nM observed for unlabeled LacI^Dimer^ S28C. Thus, LacI-Cy3 protein can be utilized to monitor the binding of individual LacI-Cy3 molecules to immobilized O_1_ DNA.

Counting of photobleaching steps indicated a mixture of singly and doubly labeled LacI-Cy3 dimer (84.8% and 15.2%, respectively) (S3 Fig).

## Conclusions

In this proof-of-principle study, we have generated a functionally active variant of LacI for a site-specific fluorescence labeling with maleimide-based fluorophores. Labeling this variant with Cy3 in the DNA-binding “headpiece” resulted in a fluorescent LacI that was able to bind to its native O_1_ operator sequence on a surface immobilized DNA, indicating that our labeling scheme is compatible with the functional activity of LacI. The fluorescently labeled LacI represents a useful tool for mechanistic *in vitro* studies of LacI target search at the single molecule level. In conjunction with an electroporation-based delivery into live cells [76], the fluorescently labeled LacI could also be used to analyze LacI search kinetics *in vivo* as the higher photostability of synthetic dyes compared to autofluorescent fusions allows the recording of longer single-particle trajectories, thus potentially revealing multiple diffusional regimes/binding modes of LacI during its target search.

## Supporting information

S1 Fig Calibration for the analytical gel filtration shown in Fig 2. The calibration of the HiLoad 16/600 Superdex 200 column was carried out with a Gel Filtration Markers Kit (Sigma-Aldrich) according to manufacturer’s instructions.
(PDF)

S2 Fig Binding curves of the LacI variants to the 5’-Cy5-labeled O1 operator DNA. Data points represent the fractional binding of the 5’-Cy5-labeled O_1_ operator DNA to LacI calculated from a quantification of the integrated band intensities. Data points were measured in triplicates, with error bars representing the standard deviation. Data points were fitted to a quadratic binding isotherm as described in the Methods section. Deviations of the fitted curve from the measured data points, in particular at low LacI concentrations, are likely due to the cooperativity observed for dimeric LacI binding to DNA that arises from the LacI monomer-dimer equilibrium [52].
(PDF)

S3 Fig Counting of photobleaching steps with LacI-Cy3. Photobleaching steps were counted in three independent replicate experiments, each with >140 binding events.
(PDF)
