# Supplementary figures and images for: Structure-guided approach to site-specific fluorophore labeling of the lac repressor LacI
Source: PLoS One. 2018 Jun 1;13(6):e0198416. doi: 10.1371/journal.pone.0198416 (PMC5983854; doi:10.1371/journal.pone.0198416)

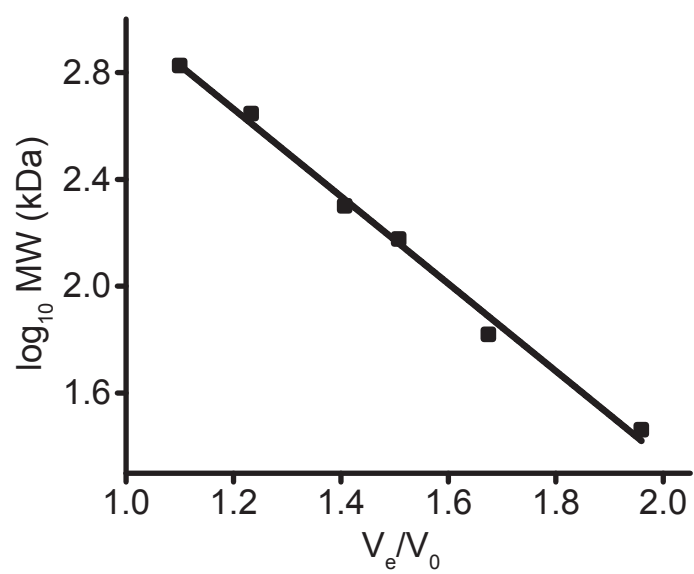

**S1 Figure, Kipper *et al.***

Supplement: S1 Fig — The calibration of the HiLoad 16/600 Superdex 200 column was carried out with a Gel Filtration Markers Kit (Sigma-Aldrich) according to manufacturer’s instructions. (PDF) [file pone.0198416.s001.pdf]

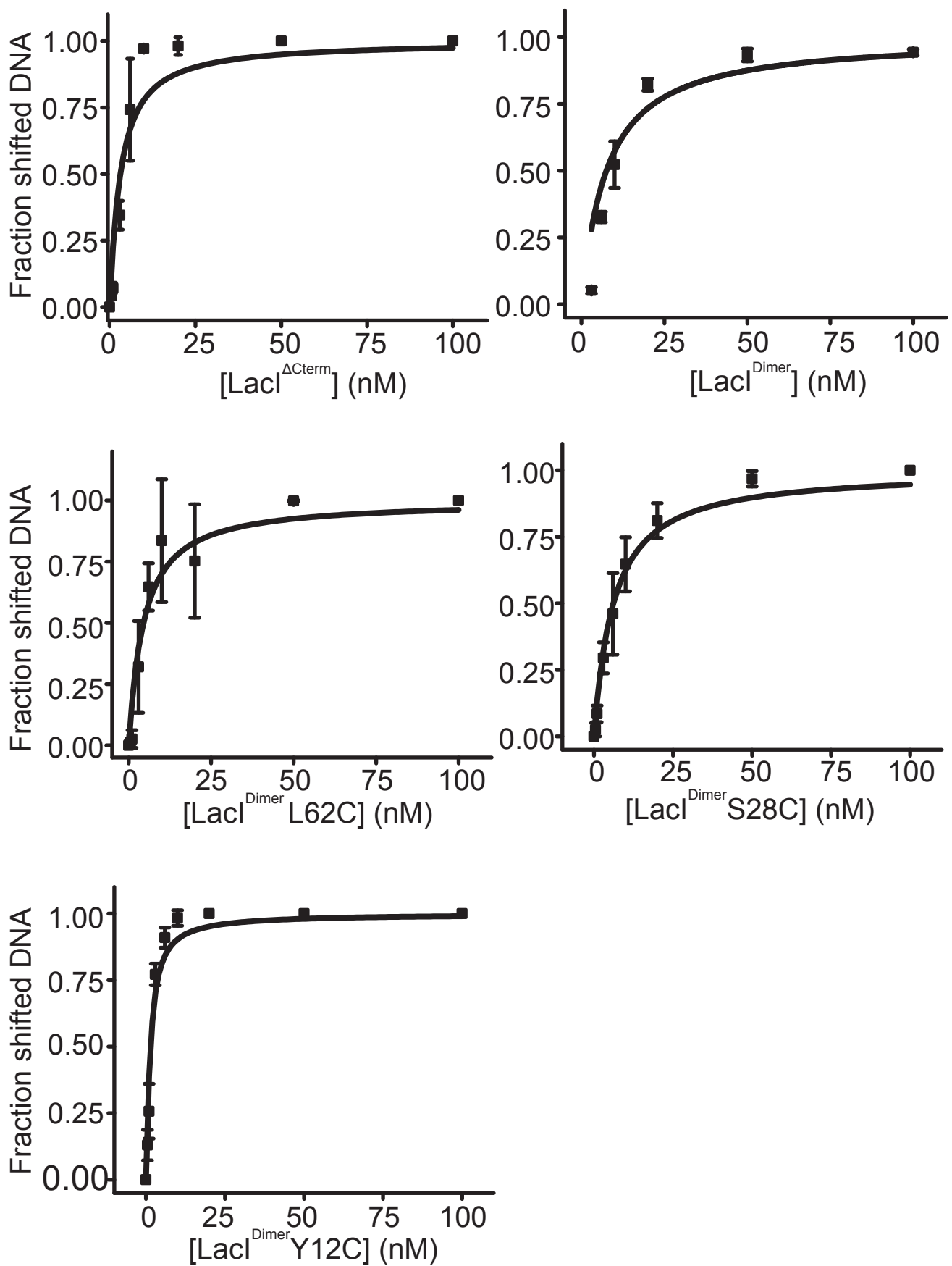

**S2 Figure, Kipper *et al.***

Supplement: S2 Fig — Data points represent the fractional binding of the 5’-Cy5-labeled O1 operator DNA to LacI calculated from a quantification of the integrated band intensities. Data points were measured in triplicates, with error bars representing the standard deviation. Data points were fitted to a quadratic binding isotherm as described in the Methods section. Deviations of the fitted curve from the measured data points, in particular at low LacI concentrations, are likely due to the cooperativity observed for dimeric LacI binding to DNA that arises from the LacI monomer-dimer equilibrium [52]. (PDF) [file pone.0198416.s002.pdf]

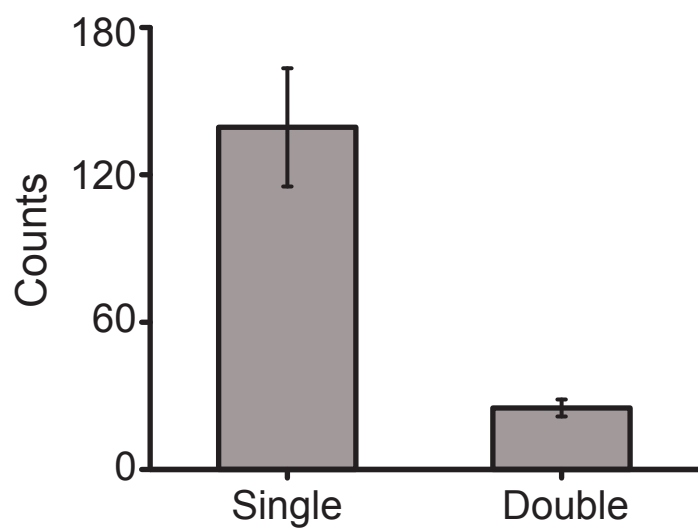

**S3 Figure, Kipper *et al.***

Supplement: S3 Fig — Photobleaching steps were counted in three independent replicate experiments, each with >140 binding events. (PDF) [file pone.0198416.s003.pdf]
